# DNA Methylation Suppresses Leptin Gene in 3T3-L1 Adipocytes

**DOI:** 10.1371/journal.pone.0160532

**Published:** 2016-08-05

**Authors:** Masashi Kuroda, Ayako Tominaga, Kasumi Nakagawa, Misa Nishiguchi, Mayu Sebe, Yumiko Miyatake, Tadahiro Kitamura, Rie Tsutsumi, Nagakatsu Harada, Yutaka Nakaya, Hiroshi Sakaue

**Affiliations:** 1 Department of Nutrition and Metabolism, Institute of Biomedical Sciences, Tokushima University Graduate School, Tokushima-city, Tokushima, Japan; 2 Research Fellow of Japan Society for the Promotion of Science, Chiyoda-ku, Tokyo, Japan; 3 Metabolic Signal Research Center, Institute for Molecular and Cellular Regulation, Gunma University, Maebashi, Gunma, Japan; 4 Cardiovascular Medicine, Shikoku Central Hospital of the Mutual aid Association of Public School Teachers, Shikokuchuo-city, Ehime, Japan; 5 Diabetes Therapeutics and Research Center, Tokushima University, Tokushima-city, Tokushima, Japan; University of Navarra, SPAIN

## Abstract

Leptin is a key regulator of energy intake and expenditure. This peptide hormone is expressed in mouse white adipose tissue, but hardly expressed in 3T3-L1 adipocytes. Using bisulfite sequencing, we found that CpG islands in the leptin promoter are highly methylated in 3T3-L1cells. 5-azacytidine, an inhibitor of DNA methyltransferase, markedly increased leptin expression as pre-adipocytes matured into adipocytes. Remarkably, leptin expression was stimulated by insulin in adipocytes derived from precursor cells exposed to 5-azacytidine, but suppressed by thiazolidinedione and dexamethasone. In contrast, adipocytes derived from untreated precursor cells were unresponsive to both 5-azacytidine and hormonal stimuli, although lipid accumulation was sufficient to boost leptin expression in the absence of demethylation. Taken together, the results suggest that leptin expression in 3T3-L1 cells requires DNA demethylation prior to adipogenesis, transcriptional activation during adipogenesis, and lipid accumulation after adipogenesis.

## Introduction

Obesity is now considered a global epidemic [1], with over 60% of adults in the United States being obese or overweight [2]. Obesity is due to an imbalance between energy intake and expenditure, and is associated with type 2 diabetes, hypertension, and other cardiovascular diseases [3]. Excess energy is stored as triacylglycerides in white adipose tissue (WAT), which further regulates energy homeostasis by secreting adipocytokines like adiponectin and leptin.

Leptin is a 16-kDa peptide hormone expressed from the Ob gene [4]. It suppresses food intake [4], and stimulates energy expenditure [5, 6]. Because circulating levels of leptin positively correlate with body mass index and fat mass [7], it is believed to be a signaling molecule that transmits signals from the peripheral to the central nervous system. In addition, leptin-deficient mice are obese, and have severe type 2 diabetes [4]. These observations highlight the need to elucidate the mechanisms that drive leptin expression in adipocytes.

Adipocyte cell lines such as 3T3-L1 or 3T3-F442A are widely used as models of adipocyte biology. These cells have fibroblast-like morphology, but they may be induced to accumulate triacylglycerides and become adipocyte-like. Nevertheless, differentiated 3T3-L1 or 3T3-F442A adipocytes express leptin at significantly lower levels than adipose tissue [8], and are insensitive to hormones like insulin [8, 9]. These properties preclude characterization of leptin expression. On the other hand, isolated primary adipocytes are challenging to manipulate genetically, or to maintain in the adipocyte state for a sufficiently long time.

Methylation, specifically of cytosines in CpG sites, is one of the several epigenetic mechanisms that suppress gene expression. Methylation generally prevents transcriptional factors from binding to promoters [10], and recruits transcriptional repressors such as MeCP2 [11]. Consequently, the expression of genes with CpG-rich promoters tends to depend on the methylation status. Indeed, the leptin promoter is CpG-rich, and methylation downregulates its activity [12]. Nevertheless, it is unclear whether methylation suppresses leptin expression in 3T3-L1 adipocytes. The aim of this study was to define the role of DNA methylation in leptin expression in cultured 3T3-L1 cells, and in obesity-induced hyperleptinemia. In addition, we wanted to establish an adipocyte cell line that expresses leptin at *in vivo* levels.

## Materials and Methods

### Cell culture and 5-azacytidine treatment

3T3-L1 pre-adipocytes, kindly provided by Dr. Hosaka at Kyorin University, Tokyo, Japan, were grown to confluence at 37°C and 7.5% CO_2_/air in high-glucose Dulbecco’s Modified Eagle’s Medium (DMEM, Sigma, MO, USA) supplemented with 10% calf serum (Thermo Fisher Scientific, MA, USA). Cells were differentiated two days after reaching confluence by replacing the media with DMEM containing 10% fetal bovine serum (GE Healthcare, Little Chalfont, UK), 10 μg/mL insulin (Wako, Tokyo, Japan), 500 μM isobutylmethylxanthine (Sigma, MO, USA), 1 μM dexamethasone (Wako, Tokyo, Japan), and 1μM troglitazone (Cayman Chemical, MI, USA). After another two days, media were replaced with 10% fetal bovine serum in DMEM, and refreshed daily thereafter. To inhibit methylation, 3T3-L1 pre-adipocytes were cultured for seven days in DMEM supplemented with 10% calf serum and 0.5–5 μM 5-azacytidine (Sigma, MO, USA). Media were replaced every other day and cells were subsequently differentiated and maintained as described. Cells were harvested at day 0 and day 6–8.

### Construction of reporter plasmids

A fragment of the mouse leptin promoter between -154 and +60 bp was amplified by PCR using mouse genomic DNA and primer pairs with sequence 5′-cagctagccgcctagaatggagcactagg-3′ and 5′-ttagatctaagactggtggaggagaaagtagg-3′ containing NheI and BglII restriction sites (underlined). The PCR product was inserted into pGL 4.19 (Promega, WI, USA) using a ligation kit (TaKaRa, Tokyo, Japan), andconfirmed by DNA sequencing.

### *In vitro* methylation of leptin promoter

The mouse leptin promoter was excised from pGL4.19 using NheI and BglII, and purified by gel electrophoresis. The insert (100 ng) was methylated with 4 U SssI methyltranseferase (NEB, MA, USA). Methylated and mock-methylated DNA fragments were then re-ligated into pGL 4.19, and purified by phenol chloroform extraction. Methylation was confirmed by digestion with SmaI, which recognizes CCCGGG, and is sensitive to methylation.

### Transfection

3T3-L1 pre-adipocytes were transfected with plasmids or siRNA using Lipofectamine 2000 (Invitrogen, CA, USA), following the manufacturer’s protocol. Briefly, 3T3-L1 pre-adipocytes were seeded one day prior to transfection in antibiotic-free DMEM supplemented with 10% calf serum. siRNA and Lipofectamine 2000 were separately diluted at the appropriate ratio in OptiMEM Reduced Serum Medium (Invitrogen, CA, USA), pooled, mixed gently, incubated for 20 min at room temperature, and added to cells. DNMT1 was silenced using siRNAs [13] with sense sequence gcugggagauggcgucaua[dT][dT], and antisense sequence uaugacgccaucucccagc[dT][dT]. Non-Targeting siRNA (GE Healthcare, Little Chalfont, UK, #D001206-14-05) was used as control. Cells were transfected with siRNA three times [13]

For reporter gene analysis, differentiated 3T3-L1 adipocytes were electroporated using NEPA 21 system (NEPA GENE, Chiba, Japan) according to the manufacturer’s protocol. Briefly, cells were trypsinized, washed in OptiMEM Reduced Serum Medium, suspended in OptiMEM Reduced Serum Medium at 50 × 10^4^ /100 μL, and transferred to a 0.2 mm gap cuvette. Cells were then mixed with 250 ng promoter plasmid, 250 ng β-galactosidase internal control, and 30 μg sonicated salmon sperm DNA (BioDynamics, Tokyo, Japan). After electroporation, 3T3-L1 adipocytes were plated in 24 well plates and cultured in 10% fetal bovine serum in DMEM for 24 h before use.

### Luciferase assay

Transfected 3T3-L1 adipocytes were harvested in lysis buffer supplied with Luciferase Assay Kit (Promega, WI, USA). The lysate was analyzed for luciferase activity according to the manufacturer’s protocol, and results were normalized to β-galactosidase activity.

### RNA isolation and reverse transcription PCR

mRNA was isolated from cultured cells or animal tissues using Trizol Reagent (Invitrogen, CA, USA), reverse-transcribed using TaKaRa PrimeScript RT reagent kits (TaKaRa, Tokyo, Japan), and analyzed by quantitative real-time PCR on a LightCycler System (Roche Diagnostics, Tokyo, Japan). Gene expression was normalized to 18S ribosomal RNA. The following primers were used: 5′-gactcccacagatggtccctac-3′ and 5′-ctgcctaaaacccctgatgagt-3′ for DNMT1; 5′-gaggactccatcacggtggg-3′ and 5′-aagaagaggcggccagtacc-3′ for DNMT3a; 5′-agccacccaagttgtaccca-3′ and 5′-acagttcccacagcgatgga-3′ for DNMT3b; 5′-agggaggaaaatgtgctgga-3′ and 5′-ggtgaagcccaggaatgaag-3′ for leptin; 5′-ggtgaagcccaggaatgaag-3′ and 5′-gaaagccagtaaatgtagag-3′ for adiponectin; 5′-ccagagcatggtgccttcgct-3′ and 5′-cagcaaccattgggtcagctc-3′ for PPARγ; 5′-ccgcagacgacagga-3′ and 5′-ctcatgccctttcataaact-3′ for 442/aP2; 5′-acggtcttcacgttggtctc-3′ and 5′-ctcaaagaaggccacaaagc-3′ for GLUT4; and 5′-agcaacgagtaccgggtacg-3′ and 5′-tgtttggctttatctcggctc-3′ for C/EBPα.

### Animal studies and adipose tissue collection

Male C57BL/6 mice were obtained from Charles River Laboratory Japan, Inc. (Yokohama, Japan). From the age of four weeks, mice were fed control diet (Oriental Yeast, Tokyo, Japan) or D12492 high-fat diet (Research Diets, NJ, USA) containing 60% fat. Animal studies were performed according to guidelines of the Animal Research Committee of Tokushima University, and were approved by the Animal Research Committee of Tokushima University. Mice were humanely sacrificed by cervical dislocation.

White adipose tissues were collected from epididymal fat pads, minced, and digested for 45 min at 37°C in PBS(-) supplemented with 2% bovine serum albumin (Sigma, MO, USA) and 2500 units/mL type II collagenase (Worthington, OH, USA). Digests were then incubated for 15 min in 10 mM EDTA, and passed through 250-μm nylon mesh. The filtrate was centrifuged at 1,200 rpm for 5 min at 4°C. Adipocytes and stromal vascular fractions were recovered from the supernatant and pellet, respectively.

### Isolation of genomic DNA and pyrosequencing

Genomic DNA was extracted from mouse tissues and 3T3-L1 cells using GenElute Mammalian Genomic DNA Mini-Prep Kit (Sigma, MO, USA), processedwith Epitect Plus Bisulfite Conversion Kit (Qiagen, CA, USA), and amplified with Pyromark Q24 PCR Kit (Qiagen, CA, USA). PCR products were used directly for analysis of DNA methylation by PyroMark Q24 (Qiagen, CA, USA), following the manufacturer’s instructions. The primers 5′-gtttagaatggagtattaggttg-3′ and biotin-5′-cctccttcttcttacctcaattt-3′ were used to amplify the leptin promoter, while 5′-attaggttgttgttgtta-3′ and 5′-taggtaggtatggag-3′ were used as sequencing primers.

### Immunoblotting

Cells were lysed in lysis buffer (20 mM Tris-HCl pH 7.4, 150 mM NaCl, 1% sodium deoxycholate, 0.1% SDS, 1% NP-40, and 2 mM EDTA). The resulting lysates were resolved by electrophoresis and transferred to polyvinylidene difluoride membranes (Millipore, MA, USA). Blots were probed overnight (14–16 h) at 4°C with primary antibodies diluted 1/500-1/1000, labeled for 1 h with secondary antibody conjugated to horseradish peroxidase, and developed with Clarity Western ECL Substrate (BioRad, CA, USA). Blots were probed with antibodies against mouse ERK (Cell Signaling, MA, USA #9101), DNMT1 (Cell Signaling, MA, USA #5119), and leptin (Santa Cruz, TX, USA #sc-842).

### ELISA

Cells were starved without serum for 4 h, and then incubated in antibiotic- and serum-free DMEM for another 24 h. The concentration of leptin in the culture media was determined by Mouse Leptin ELISA KIT (Shibayagi, Gunnma, Japan AKRLP-011).

### Statistical analysis

Data were analyzed by unpaired Student’s t test, and *p* values < 0.05 were considered significant.

## Results and Discussion

### Leptin promoter is highly methylated in 3T3-L1 adipocytes

**Fig 1:**
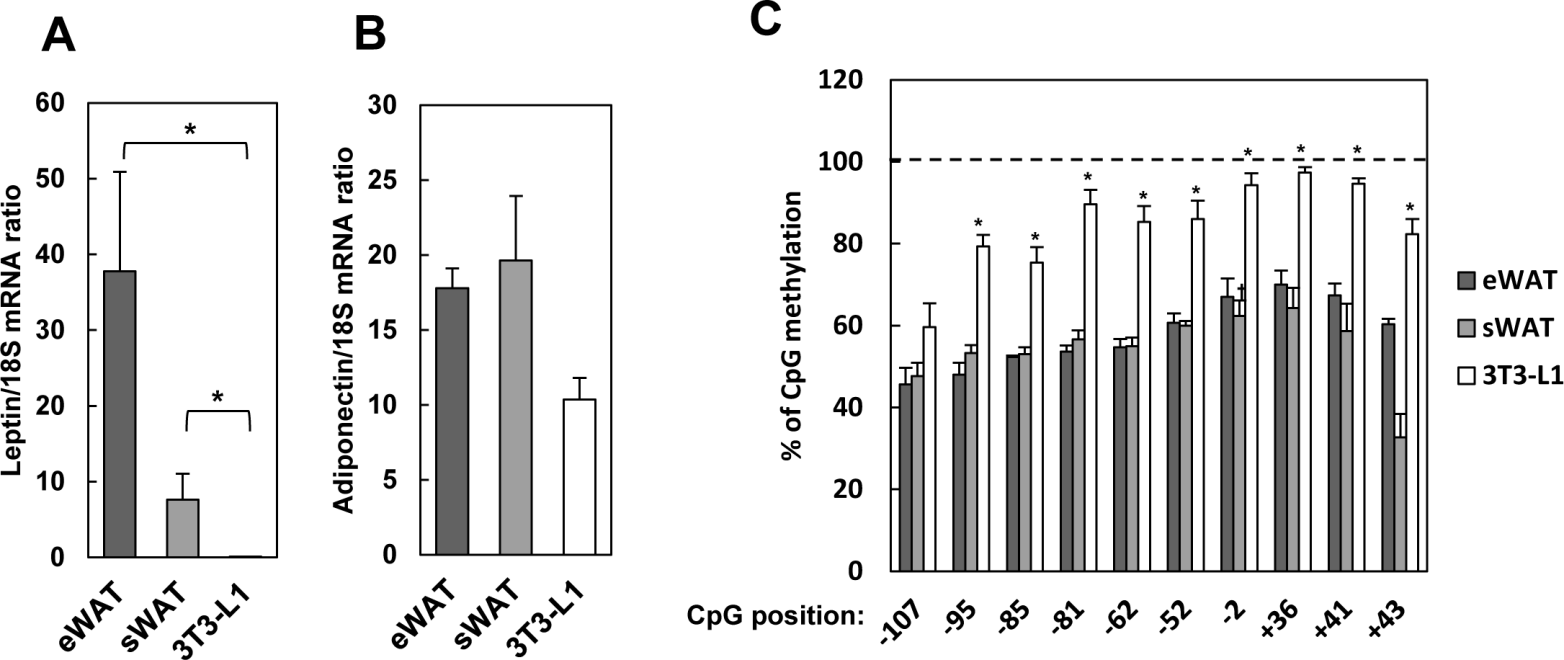
Methylation status and leptin expression. A, B: Leptin (A) and adiponectin (B) mRNA expression in epididymal (eWAT) and subcutaneous white adipose tissue (sWAT) from mice at age 12 weeks, and in 3T3-L1 adipocytes. C: Percentage of CpGs methylated in the leptin promoter in eWAT, sWAT, and 3T3-L1 cells. In A, B, C, data are mean ± SEM and *; p < 0.05, **; p < 0.01 versus eWAT.

Leptin mRNA was expressed less abundantly in 3T3-L1 adipocytes than in white adipose tissue, while mRNA levels of adiponectin were comparable (Fig 1A and 1B). We then investigated sequences -119 to +47 bp of the leptin transcription start site (S1 Fig), as this region was reported to be functionally significant [14]. We found that CpG sites in this fragment were highly methylated in cultured adipocytes than in mouse tissues (Fig 1D), implying that hypermethylation of the promoter suppresses leptin expression.

### 5-azacytidine induces leptin expression through DNA demethylation

**Fig 2:**
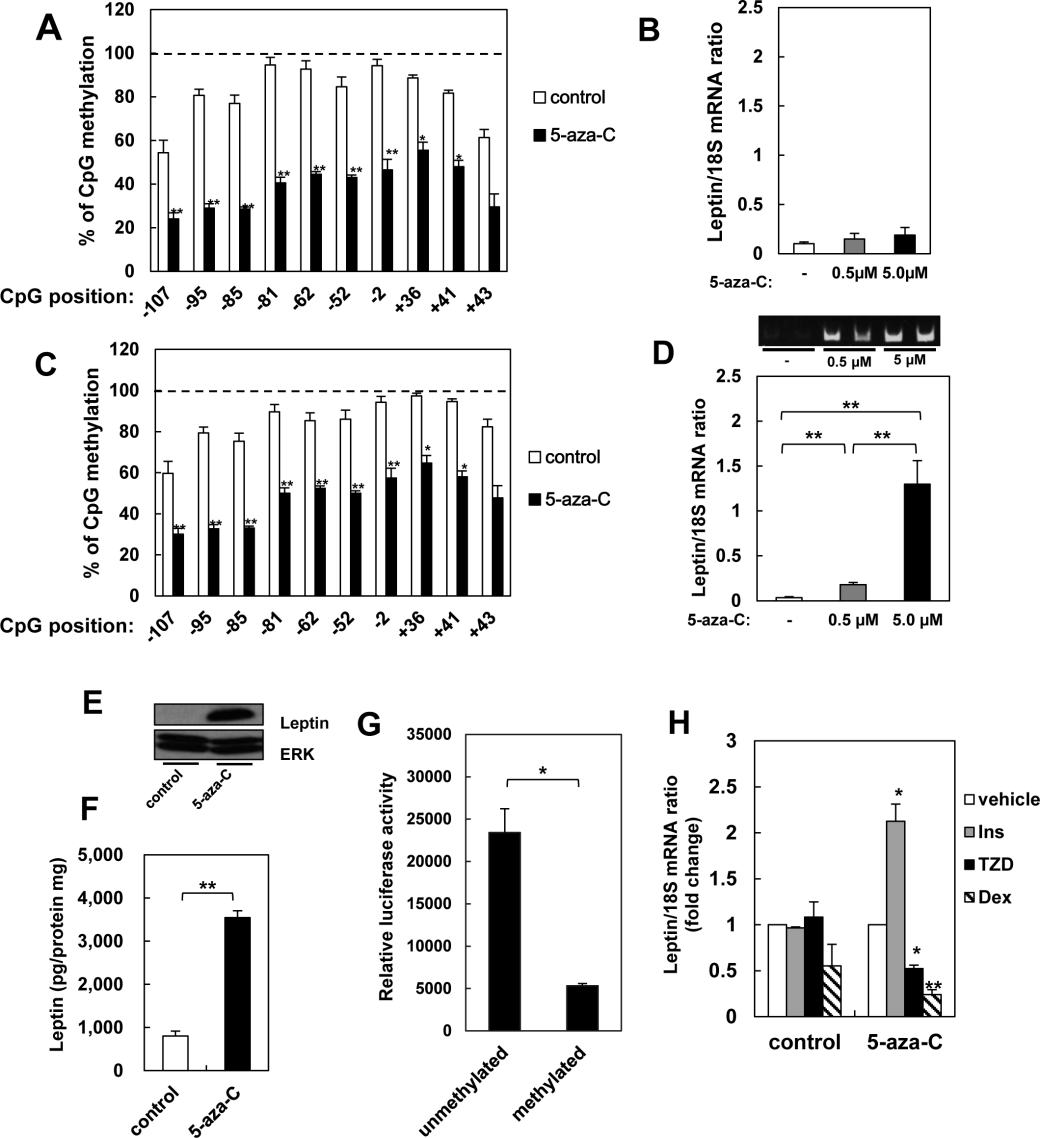
Effects of demethylation on leptin expression in 3T3-L1 cells. A, B: Promoter methylation (A) and leptin expression (B) in 3T3-L1 pre-adipocytes treated with 0.5 or 5 μM 5-azacytidine for 7 days. C, D: Promoter methylation (C) and leptin expression (D) in adipocytes derived from 3T3-L1 precursors treated with or without 5-azacytidine, measured 6 days after differentiation. Products from real-time qPCR were also separated on agarose. E, F: Expression (E) and secretion (F) of leptin in adipocytes derived from precursors treated with 5-azacytidine, as measured by western blotting and ELISA, respectively. Data were normalized to total protein concentration. G: Activity of methylated and unmethylated leptin promoter in adipocytes differentiated from 3T3-L1 cells. H: Leptin expression in serum-starved cells treated with 100 nM insulin (Ins), 1 μM troglitazone (Tro), or 1 μM dexamethasone (Dex). Cells are adipocytes derived from 3T3-L1 precursors treated with 5-azacytidine. Results are mean ± SEM. *, p < 0.05; **, p < 0.01 versus untreated cells in A-F, versus methylated promoter in G, and versus vehicle in H.

The difference in expression between 3T3-L1 adipocytes and white adipose tissue is believed to be due to methylation, which modulates the accessibility of regulatory cis-elements [10–12]. Therefore, we hypothesized that demethylation of the leptin promoter may induce expression in 3T3-L1 cells. Treatment of mature adipocytes with 5-azacytidine, an inhibitor of DNA methyltransferase [15], did not affect the methylation status and activity of the leptin promoter (data not shown). Similarly, the inhibitor did not induce expression in pre-adipocytes, although DNA methylation was reduced to levels comparable to that in white adipose tissue (Fig 2A and 2B). However, leptin expression increased 5.2- and 37.5-fold when pre-adipocytes were differentiated after treatment with 0.5 and 5.0 μM 5-azacytidine, respectively (Fig 2C and 2D). Accordingly, the amount of protein increased in cell lysates and culture media, as measured by western blot and ELISA (Fig 2E and 2F). We also directly tested whether DNA methylation affects promoter activity, and found that DNA methylation suppressed gene reporter expression by about 80% (Fig 2G).

Notably, in the present study, leptin synthesis is hormonally regulated in adipocytes differentiated from precursor cells exposed to 5-azacytidine. leptin expression in these adipocytes was stimulated 2-fold by insulin, but was significantly suppressed by dexamethasone and thiazolidinedione (Fig 2H). In contrast, adipocytes derived from untreated pre-adipocytes did not respond to these hormones (Fig 2H). Early studies suggested that leptin responds to nutritional stimuli and hormones like insulin. However, the effects of hormones on leptin transcription remain controversial. For instance, insulin was observed to stimulate leptin expression in some in vitro studies [16, 17], but not in others [8, 9] as we observed in adipocytes derived from untreated precursors.

We believe that variability in leptin sensitivity to insulin may be due to differences in DNA methylation. Methylation is considered to be a mechanism of adaptation to environmental conditions, and methylation marks are known to change dramatically in response to environmental changes, including cell culture conditions. Indeed, some in vitro studies demonstrated that DNA methylation patterns change depending on passage and other culture conditions [18, 19]. Thus, it is possible that 3T3-L1 cells insensitive to insulin, including ours, may have undergone hypermethylation, while cells responsive to insulin may have undergone demethylation.

**Fig 3:**
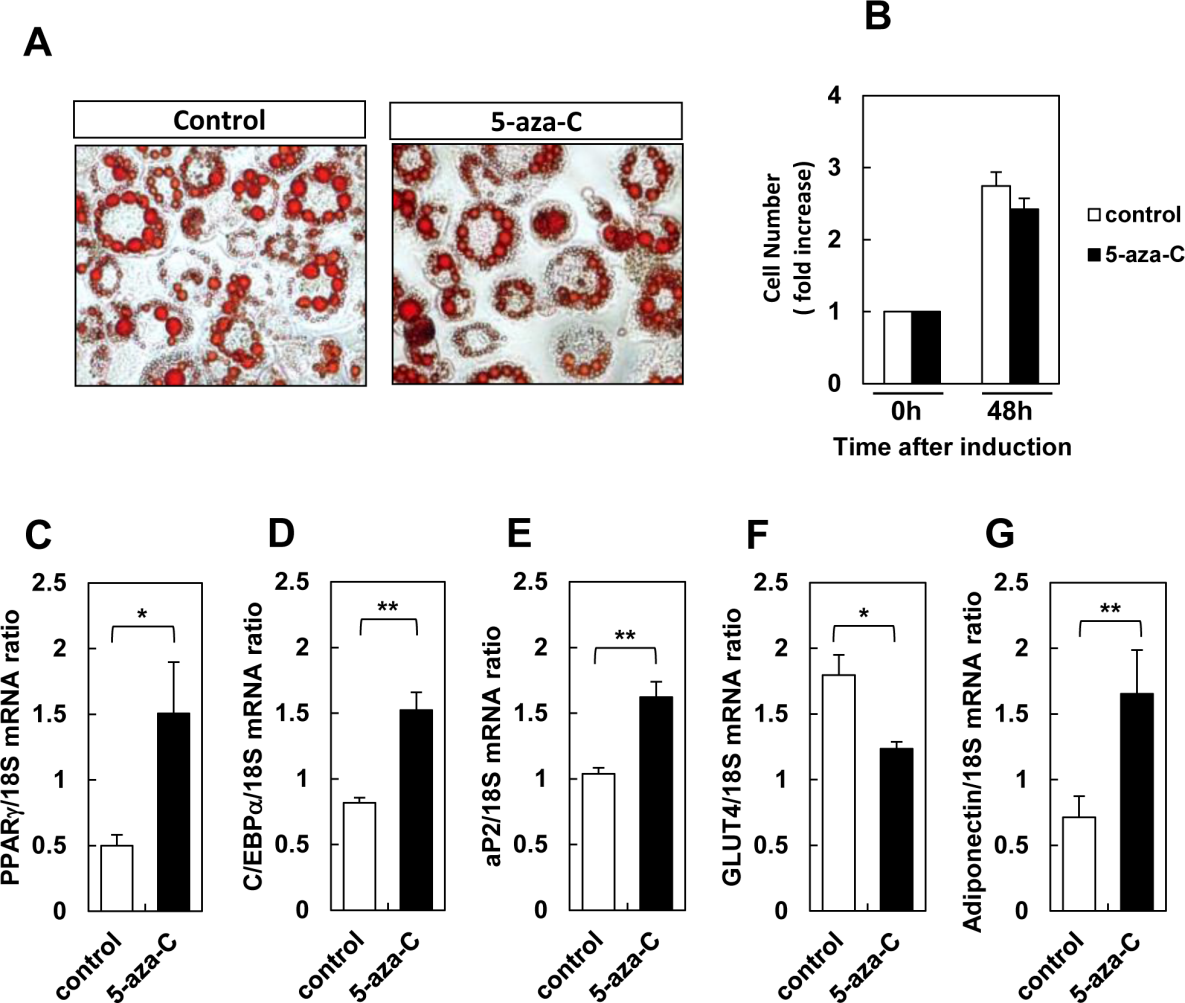
Effects of 5-azacytidine on adipogenesis in 3T3-L1 precursors treated with or without 5-azacytidine. A: Lipid accumulation, as visualized with Oil Red O in cells fixed in 3.7% formaldehyde. B: Cell proliferation. C-G: Expression of the adipocyte-specific genes PPARγ (C), C/ EBPα (D), aP2 (E), GLUT4 (F), and adiponectin (G). Results are mean ± SEM. *, p < 0.05; **, p < 0.01.

Methylation and other epigenetic marks were previously reported to regulate adipogenic differentiation[20]. Thus, we investigated the effects of demethylation on lipid accumulation, adipogenic gene expression, and mitotic clonal expansion, which are key early steps in adipogenesis[21]. 5-Azacytidine did not affect lipid accumulation and mitotic clonal expansion (Fig 3A and 3B), and the effects on adipocyte gene expression were small but measurable (Fig 3C and 3G). These results indicate that leptin expression due to prior exposure to 5-azacytidine is not exclusively due to enhanced adipogenesis.

### DNMT1 silencing induces DNA demethylation and leptin expression

**Fig 4:**
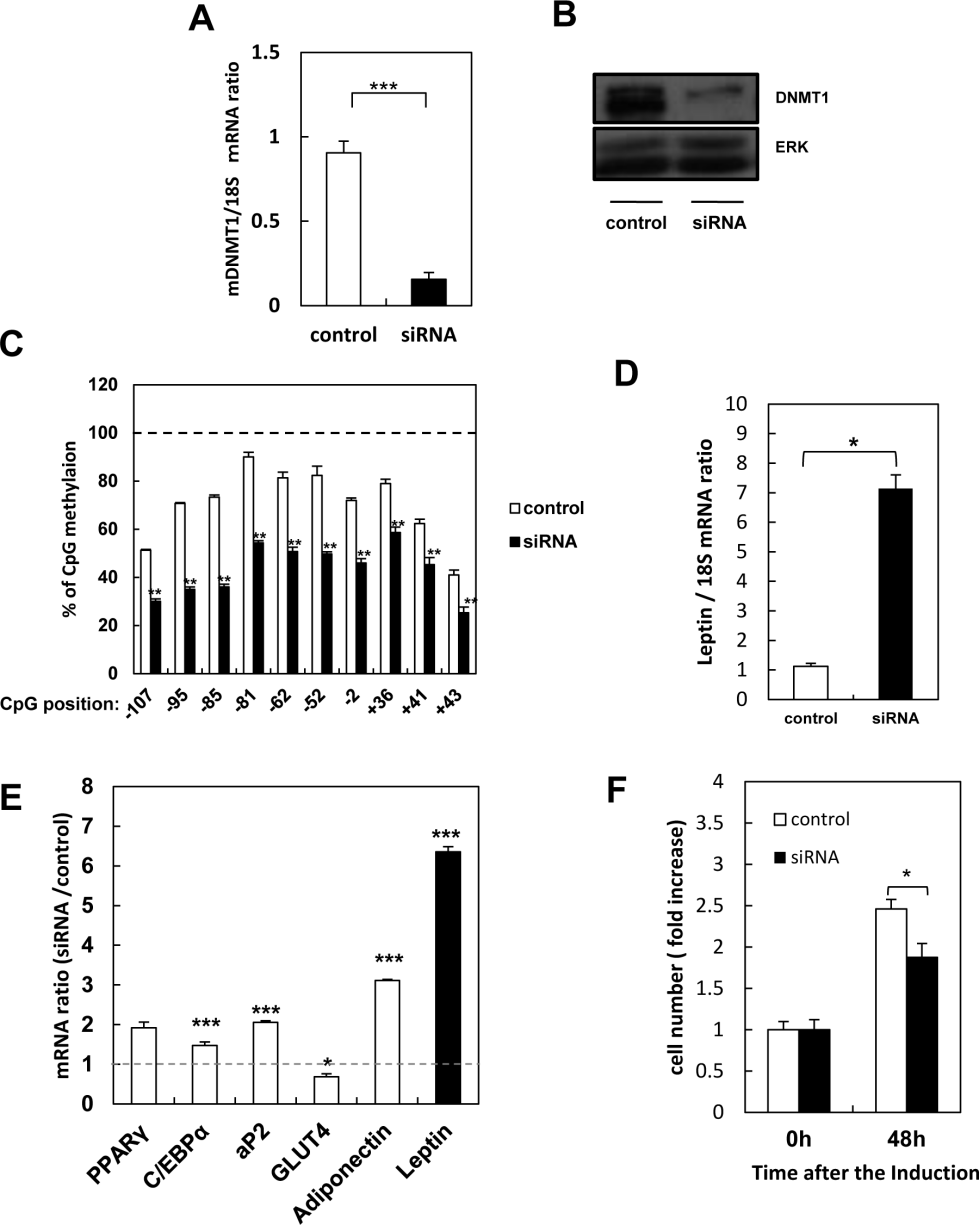
Effects of DNMT1 knockdown in 3T3-L1 pre-adipocytes. A, B: DNMT1 mRNA (A) and protein (B). C, D: Promoter methylation (C) and leptin expression (D). E: Expression of the adipogenic genes, PPARγ, C/EBPα, aP2, GLUT4, adiponectin, and leptin. F: Cell proliferation. Results are mean ± SEM. *, p < 0.05; **, p < 0.01 versus non-targeting siRNA in A-D, and versus 0 h in F.

DNA methylation patterns are established and maintained by DNA methyltransferases (DNMTs), of which three types, namely DNMT1, DNMT3a, and DNMT3b, have been identified in mammals [22]. DNMT3a and DNMT3b create new methylation marks [23, 24], while DNMT1 preserves marks established during DNA replication [25, 26]. DNA methylation was reduced in cells exposed to 5-azacytidine before differentiation. However, 5-azacytidine did not affect methylation patterns in differentiated adipocytes, strongly indicating that the inhibitor decreases methylation by inhibiting DNMT1.Therefore, we investigated the effects of suppressing DNMT1 expression by siRNA. Silencing reduced DNMT1 expression in 3T3-L1 pre-adipocytes by 80%, as confirmed by quantitative real-time PCR and western blotting (Fig 4A and 4B). Consequently, DNA methylation diminished (Fig 4C), and cells abundantly expressed leptin upon differentiation (Fig 4D), even though mitotic clonal expansion and expression of other adipocyte genes were not dramatically affected (Fig 4E and 4F). These data strongly suggest that the effects of 5-azacytidine on DNA methylation and leptin expression are due to loss of DNMT1 activity.

### Leptin expression during adipocyte hypertrophy is not due to demethylation

**Fig 5:**
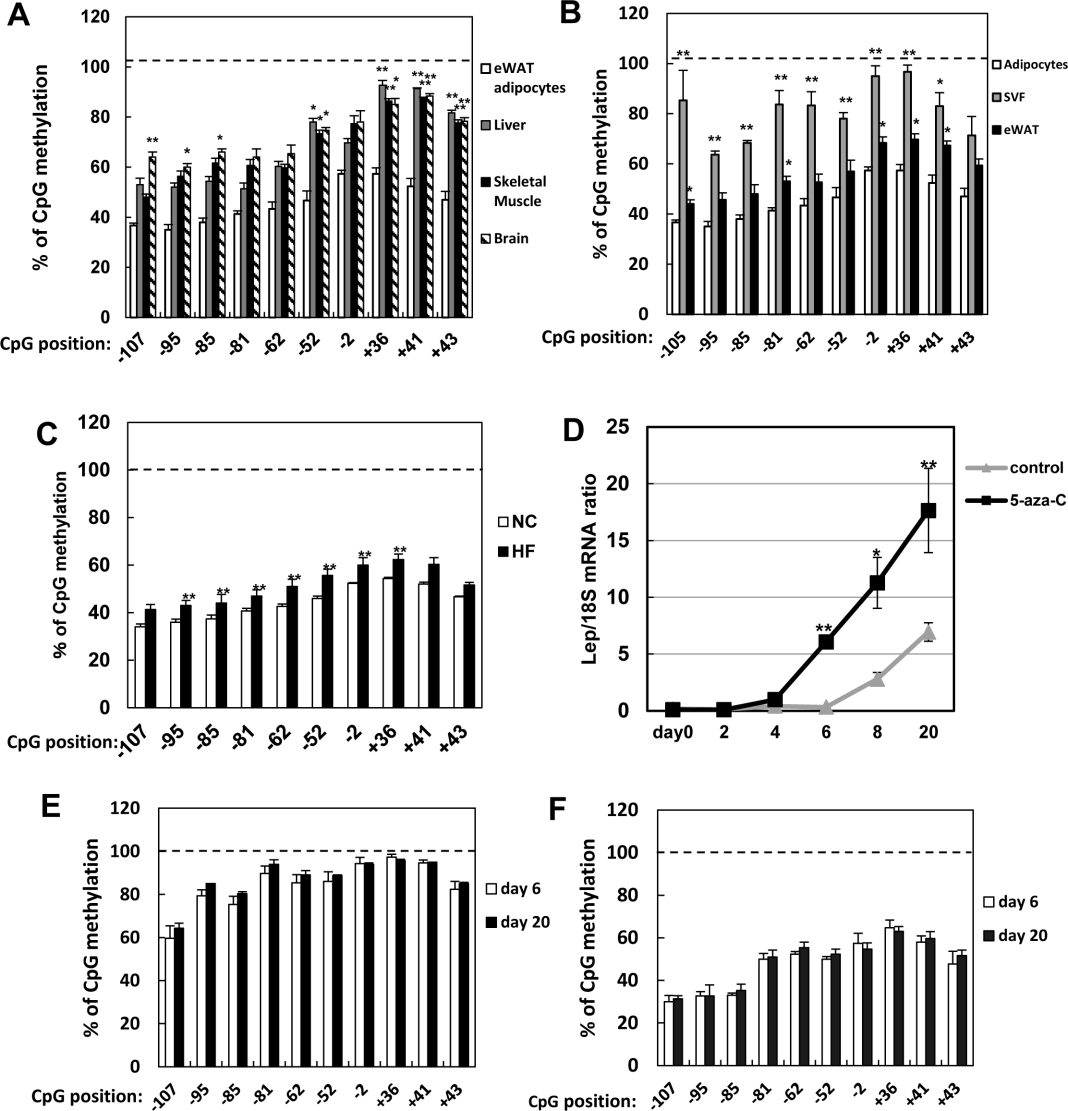
Effects of adipocyte enlargement. A: CpG methylation in eWAT, liver, skeletal muscle, and brain tissue from mice 12 weeks of age. B: CpG methylation in adipocytes and stromal vascular fraction of whole adipose tissue. C: Methylation in adipocytes isolated from eWAT of mice on normal chow (NC) or high-fat (HF) diet. D: Time course of leptin expression in adipocytes differentiated from untreated or 5-azacytidine-treated 3T3-L1 precursors. E, F: Leptin promoter methylation at day 6 and 20 in adipocytes derived from untreated (E) and 5-azacytidine-treated (F) 3T3- L1 pre-adipocytes. Data are mean ± SEM. *, p < 0.05; **, p < 0.01 versus eWAT (A), adipocytes (B), normal chow (C), untreated cells (D), and day 6 (E-F).

In rodents [4] and humans [27], leptin is primarily expressed in adipose tissues, and DNA modifications such as methylation may facilitate such tissue specificity. For instance, insulin promoters in mice and humans are uniquely demethylated in pancreatic beta cells [28, 29]. Similarly, we found the leptin promoter to be highly methylated in tissues other than white adipose (Fig 5A).

Obesity-induced hyperinsulinemia boosts leptin expression in white adipose tissues [30–32], although adipocyte hypertrophy also upregulates leptin expression on its own [33, 34]. Hence, we hypothesized that demethylation drives leptin expression during hypertrophy and we tested this hypothesis in vitro and in vivo.

Considering that whole adipose tissue consists of adipocytes and other cells with highly methylated DNA (Fig 5B), we measured methylation status only in adipocytes from mice on high-fat or normal chow diets. We found high-fat diet to slightly increase methylation of the leptin promoter (Fig 5C).

On the other hand, we induced adipocyte hypertrophy in 3T3-L1 cells by long-term culture. Leptin mRNA dramatically increased at day 6 in adipocytes derived from precursor cells treated with 5-azacytidine, and continued to rise gradually thereafter. In contrast, leptin expression became detectable beginning at day 8 in cells derived from untreated pre-adipocytes, and increased thereafter (Fig 5D). Notably, the methylation status of the leptin promoter did not change between day 6 and day 20 in both untreated (Fig 5E) and 5-azacytidine-treated 3T3-L1 adipocytes (Fig 5F). Taken together, these observations imply that methylation marks in mature adipocytes are persistent, even though precursor cells may undergo changes in methylation patterns in response to stimuli. This conclusion is inconsistent with the notion that obesity-induced leptin expression is due to demethylation. Indeed, relatively high levels persist in long-term cultures without changes in methylation, suggesting that alternative mechanisms are involved.

We did not examine variables other than adipocyte hypertrophy and obesity. For instance, dietary factors are likely to impact DNA methylation, as has been demonstrated in cases of intrauterine growth retardation due to severe nutritional deficiency. In animals, deficiency in methyl nutrients such as choline, methionine, and vitamin B12 was reported to reduce methylated cytosines in the liver [35]. Remarkably, changes in methylation were reversed upon return to a standard diet, indicating that methylation marks are dynamic, even in adult organisms.

Nevertheless, we found demethylation to be important, but not sufficient, to induce leptin release in 3T3-L1 cells. In particular, the data indicate that 5-azacytidine does not induce leptin transcription in pre-adipocytes, even though it blocks promoter methylation (Fig 2B and 2D). Indeed, leptin dramatically increases in abundance only after 6 days of differentiation *in vitro* (Fig 5D). This result suggests that the required transcription factors appear during late adipogenesis. C/EBPα is potentially one such factor, as it was reported to bind sequences -55 to -47 bp from the transcription start site [14, 36]. In mice, CpG islands in this putative C/EBPα-binding site are hypermethylated in tissues other than white adipose (Fig 5A), implying that this element is physiologically significant. However, transient overexpression of C/EBPα in 3T3-L1 adipocytes treated with 5-azacytidine did not activate leptin transcription (data not shown), indicating that it is not involved. Hence, further studies are required to identify the transcription factors that regulate leptin expression.

In summary, we found that DNA demethylation via 5-azacytidine induces leptin expression in 3T3-L1 cells that are differentiated after exposure to the inhibitor. Based on experiments with these cells, we discovered that DNA methylation before adipogenesis, transcriptional activation during adipogenesis, and adipocyte hypertrophy after adipogenesis are key events that regulate leptin expression. We anticipate that these cells will become a useful resource in investigating leptin expression.

## Supporting Information

S1 Fig Leptin promoter. Sequences -119 to +47 bp of the transcription start site in leptin are underlined. This region contains 10 CpG sites, which are highlighted in gray.
(PDF)
