# Supplementary material for: DNA Methylation Suppresses Leptin Gene in 3T3-L1 Adipocytes
Source: PLoS One. 2016 Aug 5;11(8):e0160532. doi: 10.1371/journal.pone.0160532 (PMC4975473; doi:10.1371/journal.pone.0160532)
Supplement: S1 Fig — Sequences -119 to +47 bp of the transcription start site in leptin are underlined. This region contains 10 CpG sites, which are highlighted in gray. (PDF) [file pone.0160532.s001.pdf]

CTGTTGCTGGCC**C**GCTGGGTGGGG**C**GGGAGTTGG**C**GCT**C**GCAGGGACTGGGGCTGG

-107 -95 -85 -81

C**C**GGACAGTTG**C**GCAAGTGGCACCGGGGCAGTTATAAGAGGGGCAGGCAGGCATGGA

-62 -52

GCCC**C**G<u>**G**</u>AGGGATCCCTGCTCCAGCAGCTGCAAGGTAAGGCC**C**GGGG**C**G**C**GTTAT

-2 +36 +41 +43
